# Residential greenness for mitigating impacts of extreme heat events on depression and supporting mental health

**DOI:** 10.3389/fpubh.2023.1310410

**Published:** 2023-12-07

**Authors:** Ying Yang, Yixin Zhang, Shaojie Sheng

**Affiliations:** ^1^Department of Landscape Architecture, Gold Mantis School of Architecture, Soochow University, Suzhou, China; ^2^Research Center of Landscape Heritage Protection and Ecological Restoration, China-Portugal Joint Laboratory of Cultural Heritage Conservation Science, Suzhou, China

**Keywords:** climate warming, mental illness, influence mechanisms, CiteSpace, residential greenness

## Abstract

**Background:**

Residential green spaces (RGS) are a crucial aspect of urban life, which provide residents with a positive living environment both for mental and physical well-being. However, extreme heat events caused by global warming and local urban heat island effects are threatening the public health of rapidly growing populations. This is especially true for mental health. Depression is a mental illness that can be impacted by extreme heat events, i.e., heatwaves.

**Objective:**

This study aimed to investigate the potential for residential green spaces (RGS) to alleviate depression by reducing heat stress sensitivity during extreme heat events.

**Methods:**

We conducted a literature review using scientometric analysis with CiteSpace to summarize existing research on the relationships between RGS, depression, and heatwaves. We proposed a conceptual framework for the relationship between RGS and depression, and that extreme heat events may be an important contributor to depression.

**Results:**

Our review found that RGS can provide ecosystem services that lower ambient temperatures through evaporative cooling, radiation reflection, humidity regulation, and shading. Different types of RGS, i.e., small green spaces, green roofs, green walls, and street trees, have varying cooling capacities. The mechanisms by which RGS alleviate depression during heatwaves involve green space composition, exposure, physical activity, social contacts, and cohesion. And we proposed a conceptual framework for the relationship between RGS and depression, and that extreme heat events may be an important contributor to depression.

**Conclusion:**

We present a multidimensional RGS evaluation roadmap to inform green space design for reducing depression during heatwaves. Establishing RGS multidimensional evaluation can guide future research on leveraging RGS to build resilience against extreme heat and improve public mental health.

## Highlights

- Plants provide photosynthesis for urban ecosystems; their transpiration cooling effect can regulate the local environment temperature through an evaporative process and filter air pollutants through phytoremediation, purifying the environment.
- Extreme heat episodes pose a salient risk to the well-being resulting in factors in depression.
- RGS can alleviate the impact of heat waves on mental health through climate improvement, overcoming emotional barriers, and interacting with green Spaces.
- RGS provides ecosystem services for reducing extreme heatwaves’ impact on depression.
- A conceptual framework of RGS cooling mechanisms and depression alleviation during periods of extreme heat events is put forth.

## 1 Introduction

Global warming is an urgent environmental threat facing humanity and it has a massive impact on ecosystems by sharply increasing atmospheric temperatures across the globe. Anthropogenic greenhouse gas emission increase and massive use of reflective materials in the construction of urban landscapes have led to urban heat island effects which exacerbate summer heatwaves in mid-low latitude urban areas (1–3). Rapid urbanization further changes the surface energy balance and results in further temperature rise in urban areas (4). Extreme weather events have already been severe environmental disturbances to cause devastation across every continent for the past decades, and the events’ intensity and duration are expected to increase significantly in in the near future (5). The year 2022 was the second warmest on record in Europe, and in 2023, Asia had its hottest summer on record (6). This exposure to extreme heat events has tripled in recent decades, posing a significant threat to human health, social and economic development, and ecological security.

**Figure 1:**
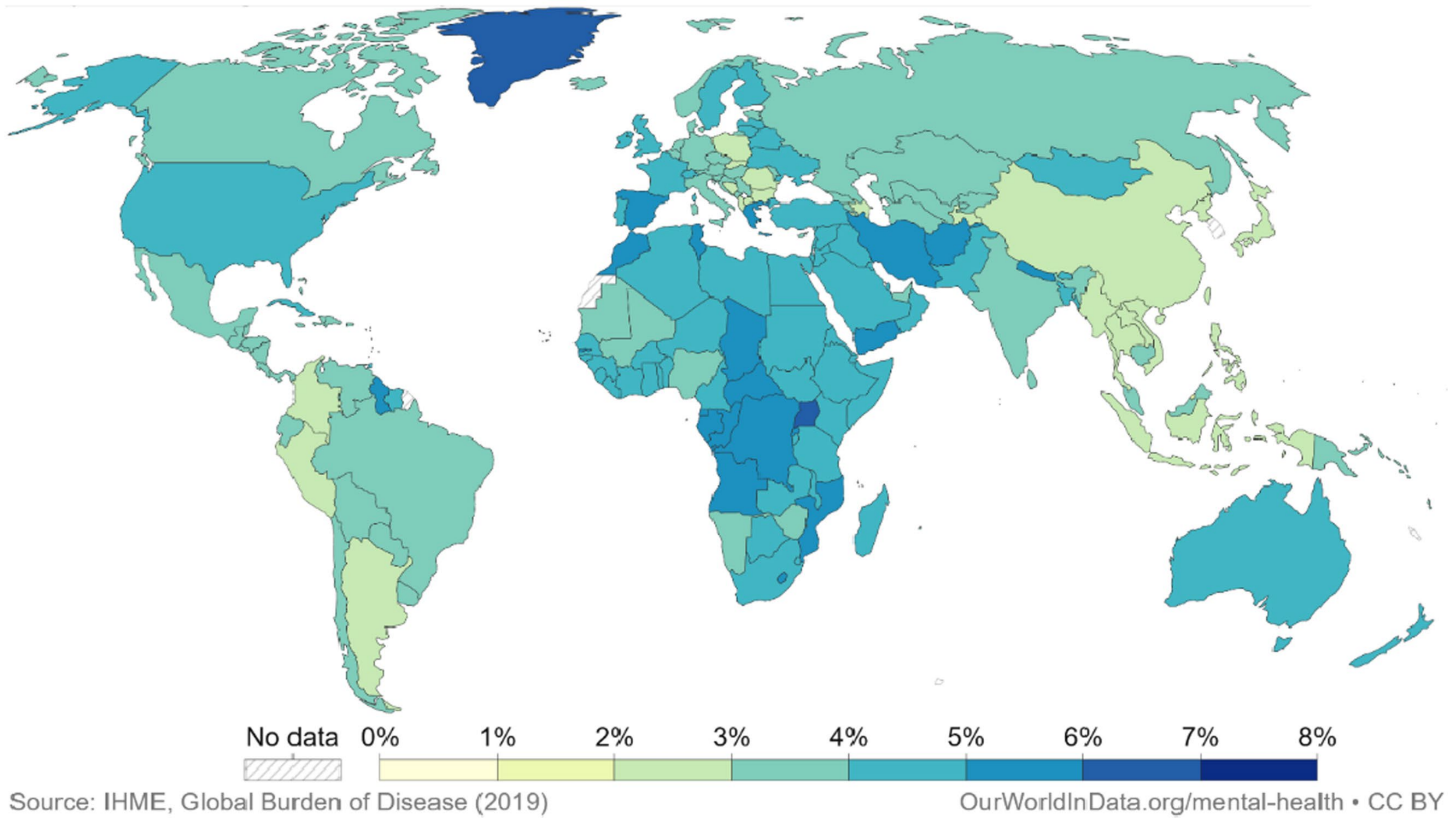
The global distribution of the population with depression, 2019. Each country is represented by a different color on the map, indicating the prevalence of depression. Lighter shades indicate a lower percentage of people with depression, while darker shades indicate a higher rate. (Source: https://ourworldindata.org/grapher/share-with-depression).

Extreme heat events caused by climate change are a fatal hazard that affects global public health (7). Extreme heat events affect human well-being in urban and rural areas through heat stress that accompanies physical, psychosomatic, and mental health symptoms by upsetting the homeostatic thermal balance of human bodies (8). The symptoms include pain in sensory organs, illnesses related to heat, fatigue, anxiety, somatic complaints, and symptoms associated with depression and cardiorespiratory issues (9). Barbosa et al. (10) demonstrated through embodied processes that mood is closely related to temperature, and exposure to heat waves negatively affects depression (11, 12). Specifically, it has direct and indirect effects (13). Some direct results include rising extreme temperatures leading to summer seasonal depression, also known as seasonal affective disorder (SAD). These cases of SAD are becoming more numerous and pronounced. Patients may find themselves experiencing symptoms such as insomnia, weight loss, and loss of appetite (14), and in severe cases, even heat-related death (15). Indirect effects include impaired mood after heat-related disasters (16) and reduced spending on health infrastructure (12). The World Health Organization (17) estimated that 4.4% of the global population was affected by depression in 2017; Ukraine, the United States, and Estonia were listed as the top three countries with the highest rates of depression globally (Figure 1). Although the factors that contribute to depression have been extensively studied globally (especially in the field of psychological research). The association of extreme weather events to residential green spaces (RGS) environment research causes a new mix of mental health risks that has not been thoroughly investigated.

Extensive research has shown that heat waves can have a negative effect on mood. Research results suggest that providing adequate green space and a more natural environment can help alleviate depression in such situations (18). We mention in this article that green spaces can be defined as any vegetated areas in urban environments consisting of trees, shrubs, grasses, vegetables, and ornamental plants. For this study, we define green spaces as parks, gardens, urban forests, greenways, community greens, and any other open spaces with abundant vegetation accessible to residents. Both public and private vegetated areas are included if they provide opportunities for passive recreation, relaxation, social connections, and other health benefits (19). However, residential green environmental exposure is considered an effective upstream urban intervention aimed at reducing the public health burden of mental disorders (20). Residential Green Space (RGS) refers to areas covered with vegetation utilized for passive or active recreation and positively impacting the urban environment, even if its impact is indirect. RGSs are easily accessible to residents and serve various needs, ultimately enhancing the overall quality of residential urban life. Exposure to and access to RGS are associated with lower levels of depression and overall mental health problems. Therefore, by creating better availability and proximity of green spaces is an effective strategy for improving mental health. Given the challenges posed by climate change, the direct and indirect cooling effects of RGS enhance people’s resistance to high temperatures and can significantly contribute to reducing depression and strengthening mental wellness health. In addition, RGS can improve social cohesion, safety, and other perceived social benefits for all users (21).

In summary, we summarize the evidence that extreme heat exacerbates depression through various physiological, psychological, and social pathways. Building on this, research on “extreme heat events,” “depression disorders,” and “RGS The current state of research suggests that access to residential green space is associated with lower levels of depression and improved mental health. In studying how RGS benefits human health under different conditions, it is critical to understand the potential of green space to alleviate depression by reducing heat stress sensitivity. This study used scientific bibliometrics tools to explore the positive effects of RGS on residents’ depression under heatwave environments, and a possible mechanism of RGS is put forward as a conceptual framework of the relationship between RGS and depression in urban heatwave environment. The framework allows for a multidisciplinary approach to show the relationship between depression and green space and suggests an optimized research path. The findings will inform urban planning and policy to incorporate residential green spaces as a strategy for building thermal-resilient and mentally healthy cities. More broadly, elucidating the mechanisms by which natural environments may confer mental health benefits under climate stress provides an ecological justification for conserving and designing biophilic spaces. This work contextualizes green infrastructure as an adaptive response to global warming within the framework of ecological public health.

## 2 Literature publication trends and analysis

To conduct a comprehensive literature review, we searched the Web of Science (WOS) (Clarivate, United States) Core Collection database for 2013–2023 using targeted keywords related to residential green spaces, depression, and extreme heat events. Specifically, we used the following search string in WOS topic fields: (“residential green space*” OR “urban green space*” OR “neighborhood green space*”) AND (depression* OR mood OR mental health) AND (“heat wave*” OR “heatwave” OR “extreme heat”). We focused our search on peer-reviewed journal articles and proceeding papers published from 2000 to 2023. After adjusting the search string through several iterations, we retrieved papers categorized as “Article,” “Proceeding Paper,” or “Review” in order to cover the full scope of research at the intersection of residential green spaces, mental health, and extreme heat.

**Figure 2:**
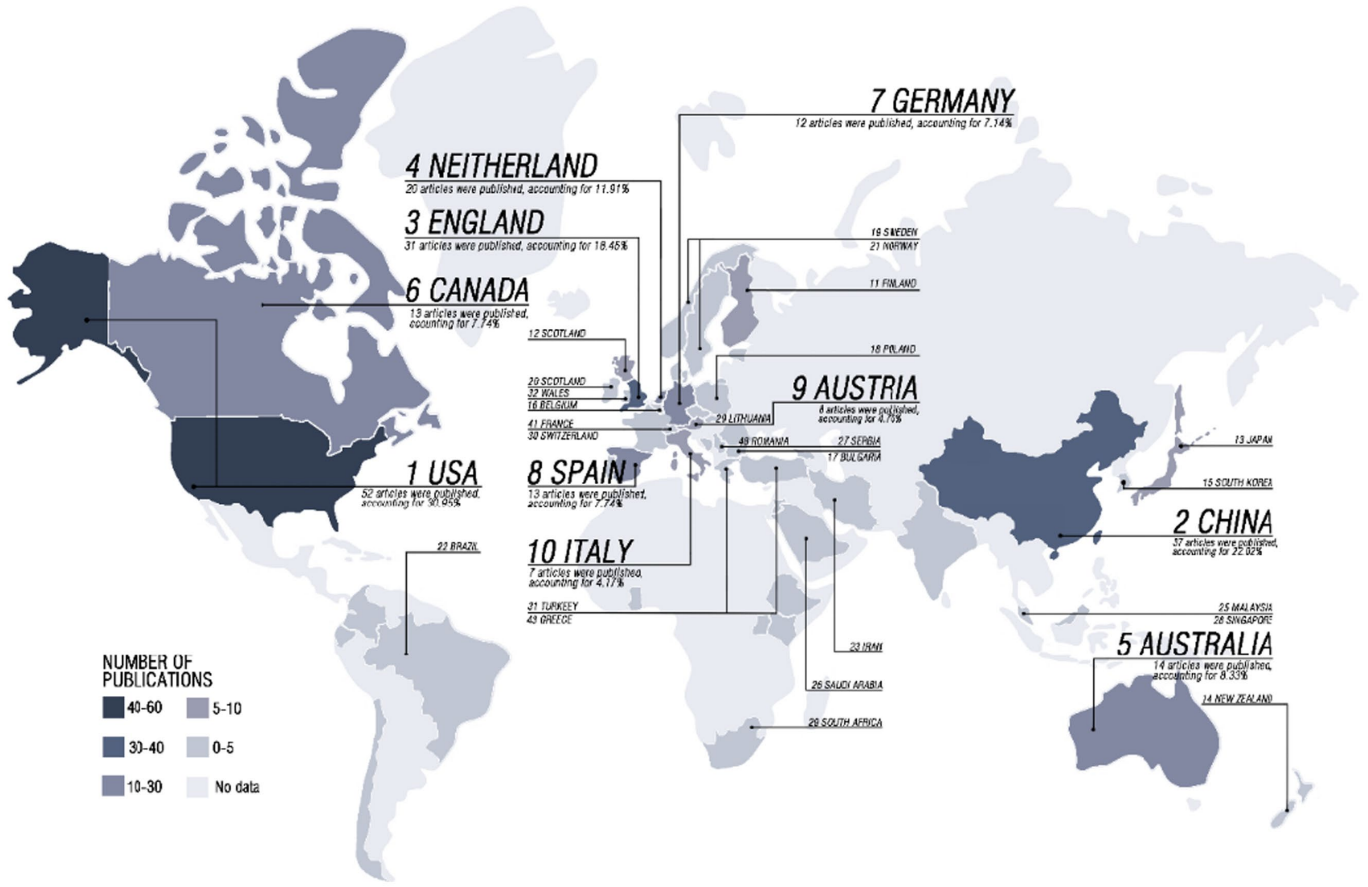
Global distribution of literature results was retrieved from 2000 to 2023.

**Figure 3:**
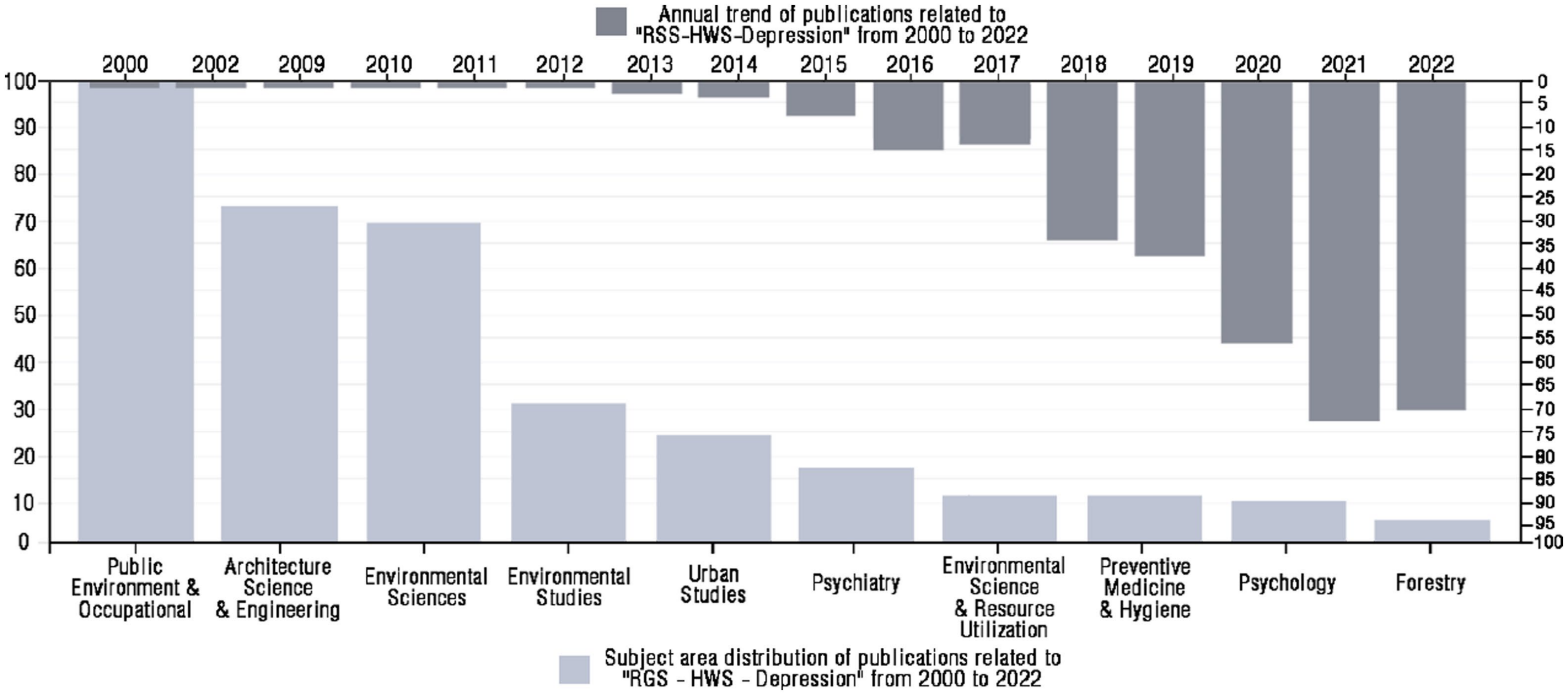
Subject areas and year distribution of the literature. The lower *X*-axis represents different disciplines, the left *Y*-axis represents the number of publications, and the light gray bar represents the number of publications related to each discipline from 2000 to 2022. The top *X*-axis is the year, the right *Y*-axis is the number of publications, and the dark gray bar is the annual number of publications from 2000 to 2022.

We leverage the bibliometric mapping capabilities of WOS, which are utilized for visualization and analysis of research trends over time, geographic distribution of publications, and subject areas covered. This allowed us to identify knowledge clusters and gaps to be addressed in this emerging field. According to the geographical distribution of global literature presented in Figure 2, the United States has published the highest number of articles in this field, accounting for 30.95% of the total. China and England followed closely, accounting for 22.02 and 18.45%, respectively. The Netherlands, Australia, Europe, and Canada also have significant contributions. Based on the number of publications and trends shown in Figure 3 that began to appear in 2000, There has been an overall trend of continuous growth since 2013 which has increased significantly in 2018. Subject distribution, public environment, and occupational health are the most published subjects.

The analysis results of the ability of CiteSpace to generate bibliometric maps show that the research mainly focuses on ecological environment, mental health, landscape evaluation, sports activities, social cohesion, social isolation and other aspects. Overall, the research topics focused on “Landscape evaluation from a public health perspective” and “Mental health mechanisms.” Given the accelerating effects of extreme weather events on mental health, there is still a knowledge conflict gap in the study of the impacts of green space on depression. Therefore, studies should integrate the relationship between RGS, extreme heat events, and depression. Overall, our search strategy and reliance on the bibliometric capabilities of WOS ensured a systematic, comprehensive literature review to inform the conceptual framework proposed later in this paper. The visualization of publication volumes, geography, and disciplines provides helpful context for interpreting the review findings.

## 3 Literature synthesis

### 3.1 Extreme heat events (heatwaves) and depression: a relationship review

Before examining the relationship between extreme heat events (heatwaves) and depression, it is necessary to define the research scope of heatwaves and depression. First, the basic definition of extreme heat events relates to a prolonged heat period of unusually high-temperature levels associated with global warming, which leads to temporary changes in people’s lifestyle and result in adverse health outcomes for the impacted populations. The definition of an extreme heat event or heatwave varies across studies, but generally refers to a prolonged period of abnormally and uncomfortably hot weather that can negatively impact health (5, 22). We focused on heatwave definitions based on absolute temperature thresholds because such fixed criteria allow for comparison across different climate regions. Currently, Tuholske et al. (23) have developed a method for defining heat waves and heat index (HI) using the formula WBGT_max_ > 30°C as a conservative threshold for extreme heat exposure based on the United States National Weather Service’s definition of overheating warnings. This may be adjusted for urban settlement exposure. It is suitable for assessing the impact of a person’s total heat exposure during a working day (up to 8 h) and for evaluating indoor and outdoor occupational environments as well as other environments. This 30°C WBGT_max_ threshold (24) is suitable for assessing total heat exposure during daylight hours and across different environments. We chose this definition because it is tailored to evaluating heat impacts on human health and occupational activities, which aligns with our focus on the public health implications of heatwaves (25). Standardizing heatwave definitions allows for consistency in research on health outcomes like depression. We selected recognized absolute temperature criteria to enable generalization and integration of findings across geographic regions with different climates. This can help advance understanding of heat-health relationships globally.

Then, the World Health Organization defines depression as a mental disorder characterized by long-term sadness, low mood, or a lack of interest or pleasure in activities (26). Depression is caused by social, psychological, and biological factors, and those who have faced adverse life events or disasters are more vulnerable to developing it. Conversely, depression can exacerbate stress levels and functional impairments further aggravating the patient’s living conditions and depression (14). By defining extreme heat events, it can ensure that researchers use the same definitions and standards making the research results more reliable and comparable. Furthermore, defining depression can help researchers determine whether study participants meet the clinical diagnostic criteria for depression avoiding misdiagnosis related to mood disorders other than depression. Ding et al. (27) examined effects of extreme heat events on mental health in Australia. That research focused on the response relationship between extreme heat events and mental illness (anxiety and depression) (28–30). This included the diagnosis rate of depression (31), mortality rate (32), etc. Furthermore, some studies have found a positive association between extreme heat events and depression. A possible explanation for this may be that heat stress disrupting thermoregulatory mechanisms causes physiological changes in the body, such as dehydration and electrolyte imbalances, that affect mood and cognitive function (33) and can disrupt sleep (34), a known risk factor for depression (35). In addition, extreme heat events leading the pressure to depression can significantly have impacts on social and economic aspects. For example, extreme heat can lead to reduced work efficiency, which can lead to financial hardship and unemployment. Thus, extreme heat events have substantial societal costs of lost productivity. For example, 295 billion work hours were lost in the year 2020 worldwide (about 88 work hours per employed individual), due to extreme heat events exposure (36). This results in stress and anxiety, known risk factors for depression (26).

The potential correlation between extreme heat events and depression is complex and not fully understood. However, some key evidence indicates heatwaves can negatively impact mental health (37). For instance, studies have shown associations between high temperatures and increased emergency room visits for mental illnesses, including mood and anxiety disorders, schizophrenia, and stress reactions. Additionally, higher rates of depression symptoms and reduced mental wellbeing have been reported in populations exposed to extreme heat events compared to normal summer temperatures. Further research is still needed to unravel the multifaceted relationships between heat stress and various mental health outcomes like depressive disorders. From the perspective of RGS, further research is needed to elucidate potential mechanisms to mitigate the possible adverse effects of extreme heat events on mental health.

### 3.2 RGS and extreme heat events: ecosystem services perspective

**Figure 4:**
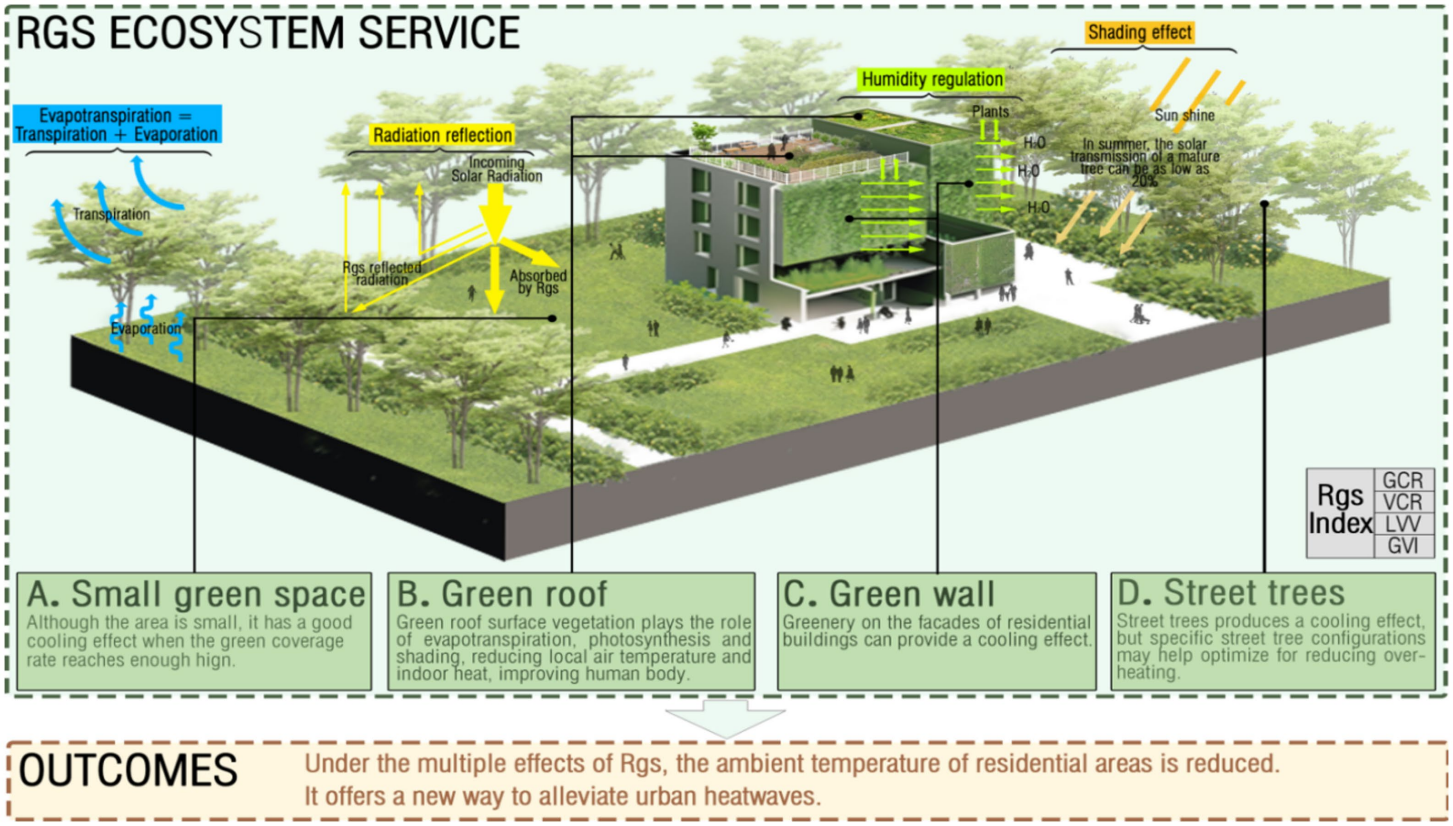
Cooling effect of RGS and mechanisms of alleviating extreme heat events. The figure shows that the RGS provides ecosystem services to reduce the ambient temperature from four aspects: evapotranspiration, radiation reflection, humidity regulation, and shading effect. In addition, different types of RGS play different cooling roles.

RGS systems are a highly active and relatively independent subsystem within urban ecosystems, which reflects intrinsic characteristics and attributes that provide health benefits (38). Previous studies have looked at the types of RGS include small green spaces (39), green roofs (40), green walls (41), and street trees (42), as well as the effects of RGS characteristics such as greening rate and structure on extreme heat events (Table 1). Some studies have found that the spatial distribution (43), structure (44), and higher greening rates of different types of green space and RGS can effectively alleviate the impact of extreme heat events. RGS can mitigate extreme heat events by ecosystem services, such as regulating temperature, humidity, and wind speed (45, 46). These ecosystem services can be achieved by increasing vegetation cover and improving ecosystem structure and biodiversity (47, 48) in addition to strengthening the health and resilience of urban ecosystems, thereby reducing the impact of extreme heat events. The processes of cooling the surrounding environment through green space are linked with four main mechanisms: evapotranspiration, radiation reflection, humidity regulation, and shading effect (Figure 4). Plants release water through transpiration and evaporation, which lowers temperatures. The surface of the green space reflects sunlight and radiant heat. Green space can also change the wind direction, increase airflow and circulation, adjust humidity, and create a comfortable living environment. In addition, green roofs and walls also provide insulation, effectively reducing indoor temperatures. Therefore, this section summarizes the effects of RGS ecosystem services on the residential environment temperature and depression from four aspects: green space types, structure, and corresponding measurement index (green coverage ratio, vegetation coverage ratio, living vegetation volume, and green view index).

**Table 1:** Study the cooling and depression relieving effects of the RGS study type under heatwave impact.

Study type		Cooling effect	Depression relieving effect	Reference
Green space types	Small Green Space (SGS)	The larger the green area and vegetation coverage, the more pronounced the cooling effect.	A place to socialize, rest, and psychological recovery.	Oliveira et al. (45), Yan et al. (50), and Nordh and Østby (52)
	Green Roof (GR)	GR can reduce temperatures by 17 to 28% compared to regular roofs.	GR increases heat comfort and indirectly improves mental health.	Zinzi and Agnoli (54), Currie and Bass (55)
	Green Wall (GW)	The maximum surface temperature difference between GW and bare wall can reach −20°C.	GW can buffer the negative psychophysiological consequences of stress.	Mazzali et al. (41), Chan et al. (57), and Gunn et al. (58)
	Street trees	Evenly distributed street trees may minimize air temperature and radiative cooling.	Street trees reduce depression and promote social justice.	Morakinyo et al. (60) and Marselle et al. (61)
Green Index	Coverage ratio	Green coverage ratio (GCR) and vegetation coverage ratio (VCR) differences are the leading cause of the UHI effect, negatively correlated with surface temperature.	GCR and VCR were inversely associated with the prevalence of depression.	Hulley et al. (62), Tian et al. (63), and Abdullah et al. (64)
	Living vegetation volume (LVV)	The cooling effect of green space is affected by its spatial structure, and the difference is reflected in LVV.	Green space structures were inversely associated with incidence.	Wu et al. (68), Reyes-Riveros et al. (70), and Chang et al. (71)
	Green view index (GVI)	A higher GVI indicates more green coverage and better ecosystem services.	Residents living in a high GVI environment have a lower risk of depression.	Zhao et al. (72) and Braçe et al. (73)

#### 3.2.1 Effect of different types of RGS

##### 3.2.1.1 Small green spaces (SGS)

The area of residential SGS is generally 0–0.5 ha (49). Green space area significantly impacts the cooling effect and coverage range with larger green areas resulting in lower air temperature (50). Studies show that even a green space area of only 0.24 ha with a 96.5% vegetation cover can lower temperatures by 5.5–6.2°C compared to surrounding streets (45). In regards to mental health, SGS provide protection and promotion mechanisms (51) primarily for socializing, resting, and spiritual recovery (52).

##### 3.2.1.2 Green roofs (GR)

GR have become an essential solution for cities to cope with extreme heat events. These structures can effectively isolate UV rays and improve the thermal comfort of buildings by utilizing the evapotranspiration, photosynthesis, and shading effects of vegetation on the GR surface (40) to potentially enhance citizens’ quality of life and indirectly improve their mental health (53). Zinzi and Agnoli (54) showed that GR can reduce the temperature by 17–28% by greening the roof compared to ordinary roofs. A 10–20% increase in green roof areas will significantly contribute to all citizens’ social, economic, and environmental health (55).

##### 3.2.1.3 Green walls (GW)

GW significantly impact the cooling effect of street canyons and buildings (56). Mazzali et al. (41) measured that the maximum surface temperature difference between GW and bare walls could reach −20°C. In some studies, GW are classified as urban green infrastructures to discuss the impact on health, and vertical greening outside buildings can buffer the negative psychophysiological consequences of stress (57). Gunn et al. (58) concluded that natural elements’ emotional and health benefits decreased after 2–5 weeks of exposure by studying houseplants implemented as green walls and natural landscapes in schools.

##### 3.2.1.4 Street trees

Planting native-species trees along streets in residential areas is a type of nature-based solutions (59). Evenly distributed street trees on all sun-exposed street locations in a neighborhood or community may be the most effective way to reduce extreme heat events and maximize air temperature cooling and radiation cooling (60). In a study of the relationship between the number and type of street trees, the distance residents lived from homes, and the number of antidepressant prescriptions, it was found that the more trees around the house (< 100.0 m), the lower the risk of taking antidepressants (especially in disadvantaged groups). Street trees may also reduce the health inequalities gap between economic and social groups (61) and promote social equity.

#### 3.2.2 Green index for RGS

##### 3.2.2.1 Coverage ratio

Generalized greenness (including green coverage ratio [GCR] and vegetation coverage ratio [VCR]) is an important index to evaluate the quality of the living environment. Studies have shown that the difference between GCR and VCR is the leading cause of the urban heat island effect (62, 63). Studies have shown that healthy vegetation cover negatively correlates with psychiatric and non-psychiatric diseases (64). Another study showed a negative correlation between green coverage and the incidence of depressive symptoms in middle-aged and older adult individuals living in urban areas (65). Most of the available findings related to human benefits associated with reducing depression are related to generalized greenness [typically captured by normalized vegetation index (NDVI)] (66). Areas with high NDVI values typically indicate good environmental quality, improved air quality, and better human health. Liu et al. (67) studied the relationship between NDVI and depression and showed that every 0.1 unit increase in NDVI would lead to a significant negative correlation of 7.0% depression. Therefore, NDVI can indicate the influence of residential green spaces on the mental well-being of inhabitants.

#### 3.2.3 Living vegetation volume, the cooling efficiency of RGS

The Living Vegetation Volume (*LVV*) is affected by its spatial structure (68) and the LVV can better reflect the difference in green space structure. It more intuitively reflects the ecological benefit potential of green space vegetation at the level of vegetation ec0logical function. In the range of 400.0 m^2^, the LVV needs to reach 200.0 m^3^ for green space to play a cooling role. When the LVV per unit area is more significant than 1.0 m^3^/m^2^, the cooling effect of green space is noticeable, and the maximum reduction is 1.5°C (69). Studies related to health are mainly associated with green space structure and show strong evidence of positive effects (70). There are few studies on green space structure and depression, but there are studies on the correlation between schizophrenia and green space structures. Most indicators of green space structure, including average patch area, edge density, and higher complexity of green space, are negatively correlated with the incidence of schizophrenia, which may reduce the risk of schizophrenia (71).

##### 3.2.3.1 Green view index

The Green View Index (GVI) is an indicator used to evaluate urban green coverage and ecosystem services, with a higher GVI indicating more green coverage and better urban ecosystem services. Studies have shown that GVI relates to residents’ physical and psychological health, social interaction, and housing satisfaction. For example, a higher GVI is associated with lower rates of mental illness, and people in environments with a higher GVI tend to be calmer and more peaceful. However, the GVI directly reflects the level of ground plants, wall greening, and other green plants. Generally, areas with higher GVI have higher greening coverage, and the actual number of vegetation increases. The greening index can be improved from a two-dimensional to a three-dimensional level through the GVI (72). A cross-sectional study was conducted by Braçe et al. (73) to evaluate the impacts of GVI on depression, and the results showed that adults living is areas with higher GVI had a lower risk of depression. People who enjoy green space from home have a lower risk of anxiety and depression.

### 3.3 RGS and depression: the health perspective

From the perspective of green space types and indicators, the effects of temperature regulation services of RGS ecosystem services on depression were summarized in Table 1 and show that different green space types had an impact on depression relief and were negatively correlated with green indicators. In another study conducted in Finland, individuals living in areas with a high degree of residential greening were at a lower risk of depression with the extent of the effect influenced by factors such as the type of depression being evaluated, the quality of the spatial data used to measure residential greening, and the spatial scale of the analysis (74). Overall, RGS exposure is negatively correlated with depressive symptoms (75); while there’s no definitive data on how much green space can alleviate depression. Research data suggest that a 10.0% increase in green space is associated with a 3.0% reduction in depression risk. On the other hand, the effectiveness of RGS ecosystem services is also reflected in improving overall mental health (76, 77). This includes increasing life satisfaction (78), increasing happiness and subjective well-being (79), improving daily life (80), enhancing sleep quality (81), and relieving stress (82), as well as improving anxiety, attention deficit, and hyperactivity disorder (83), etc.

In addition, the mechanism by which green spaces alleviate depression may be related to individual and social factors such as sports activities, social contact and cohesion, recovery and stress relief, gender, education level, and socio-economic status (84). Green spaces can help regulate the risk of depression by providing an environment for physical activity, exercise, and opportunities for social activities that can promote interpersonal communication and community participation (85). Social contact and cohesion (84) with increasing green spaces have been shown to reduce the risk of depression by strengthening community solidarity and a sense of commonality. Different age groups and genders have different degrees of susceptibility to extreme heat conditions. Vulnerable groups such as children, adolescents, and older people are more sensitive to temperature changes, while young and middle-aged adults are more heat resistant. Regarding gender differences, men are more influenced by environmental factors than women (86). In a study of people with low education and lack of natural experiences in childhood (87), access to green spaces improved mental health significantly. Green Spaces can also provide an egalitarian social and communicative environment that strengthens social support and connections. This is especially true for those of lower socioeconomic status who are more likely to suffer from depression (88).

### 3.4 Summary

**Figure 5:**
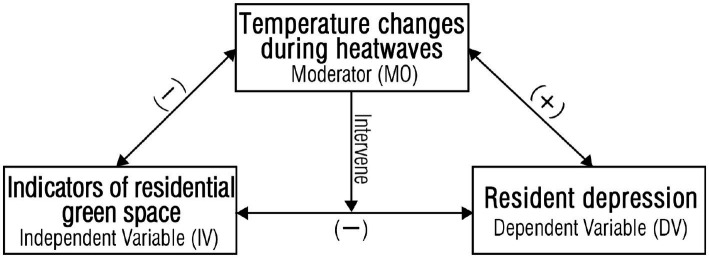
Variable factors in the study design of RGS and depression under extreme heat events.

The literature reviewed demonstrates negative correlations between various RGS indicators and high temperature/heatwaves, as well as positive associations between heat and depression (Figure 5). Specifically, key findings from original research to date on RGS, extreme heat events, and depression include: (1) Observational studies finding higher rates of mental health issues like depression, stress, and anxiety during or following heatwaves compared to normal summer temperatures. This indicates heat is a risk factor for mood disorders; (2) Clinical studies revealing possible biological mechanisms such as heat stress-induced changes in hormones, neural chemistry, cytokines, hydration, and sleep that may underpin heat-related mood impacts; (3) Epidemiological analyses showing residential proximity and access to urban green spaces is associated with lower rates of depression and antidepressant prescriptions; (4) Experimental studies demonstrating RGS can significantly reduce ambient temperatures through multiple cooling mechanisms (evaporative cooling, shading, etc.) compared to non-green built areas; (5) Initial research finding certain RGS characteristics like vegetation cover and structural complexity may enhance cooling capacities and be relevant to mitigating heatwave health risks. This aligns with other studies showing links between extreme heat, worsened mental health, and the cooling capacities of urban greenery (89–91).

**Figure 6:**
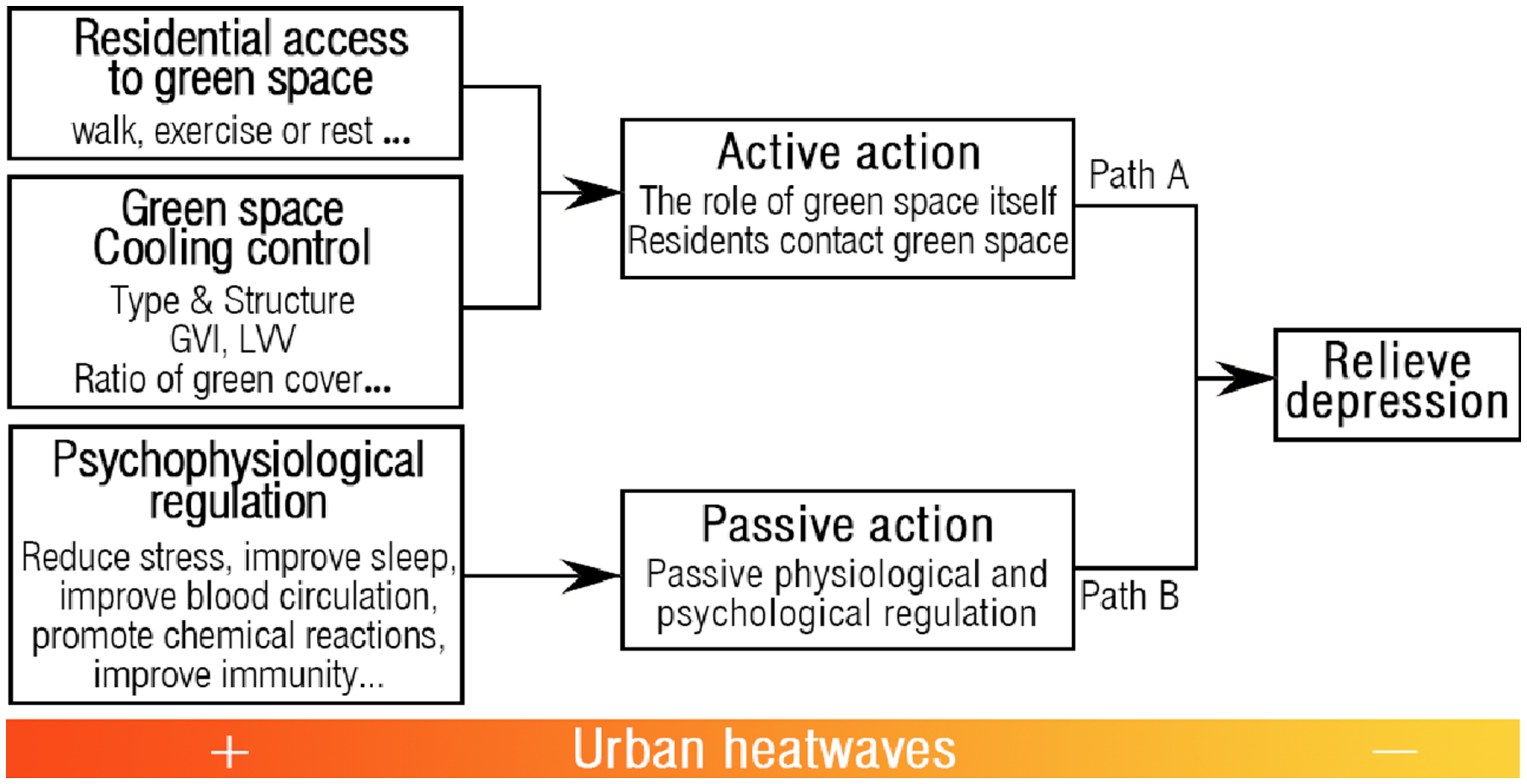
Mechanisms through which depression may be alleviated by residential green spaces reducing effects of extreme heat events.

So we have identified a mechanism through which depression may be alleviated by residential green spaces reducing the effects of extreme heat events. The various indexes of RGS were negatively correlated with high temperature and depression during heatwave impacts. At the same time, depression was positively associated with temperature. This was also supported by other studies. The process of providing ecosystem services through RGS involves a variety of pathways that are beneficial to mental health. These include the active cooling effect of green space and the activation of patients’ self-activity in contact with green space, as well as the passive regulation of psychology, as shown in Figure 6.

The details are as follows: (i) RGS active temperature regulation is one of the main bodies on the supply side and affects the health status of community residents (92). Their health status is affected by a variety of factors inclusive of vegetation type characteristics (93), other characteristics (size, type, configuration, and other qualities) (94), management and supervision (95), etc. The cooling effect of green space is due to the flow of relatively cooler air from the green space system to the non-green space system (96), and the size and spatial layout of green space will affect the cooling effect (97). During the hot summer months, green spaces can provide shade and cooling effects, alleviate effects of extreme heat events, and reduce residents’ heat stress and disease risk; (ii) Various types of contact between residents actively and RGS can improve depressive symptoms (98). The community is one of the most frequent places where residents live and engage in social activities. Research has demonstrated that outdoor landscapes in residential areas can enhance the physical health of older individuals through activities like socializing with friends, sports, and observing green spaces (99). Walking, exercising, or resting in a green space can reduce the body’s stress response, relieve stress and tension, improve mood, and release endorphins, a chemical that can improve mood; (iii) Passive temperature regulation of RGS targeting physiological and psychological regulation. The body is more prone to excitement and anxiety in high temperatures. Also, blood pressure rises. In addition, high temperatures can significantly affect sleep quality or cause a lack of sleep, and overexertion can exacerbate depression. Long-term exposure to high temperatures is associated with depression (100). In this case, green spaces can provide shade and cooling effects providing residents with a cool and comfortable environment, thereby reducing their heat stress and improving sleep quality as well as alleviating depressive symptoms (100).

## 4 Perspective: practical approach of RGS and depression under heatwaves

The purpose of this section is to propose a methodology to empirically test the conceptual framework linking RGS, heatwaves, and depression presented earlier. A clear roadmap can guide future research to evaluate the relationships and mechanisms hypothesized in the framework.

### 4.1 The conception framework of RGS for relieving depression under heatwaves

The utilization of RGS to alleviate depression during extreme heat events has not been thoroughly investigated via systematic research. Therefore, it is necessary to summarize and systematically describe the conceptual framework (77, 101). The role of RGS in cooling and humidifying different types of green space, ventilation, and reducing thermal radiation can improve the environmental heat wave in residential areas. Then the contact between residents and RGS can jointly relieve depression.

**Figure 7:**
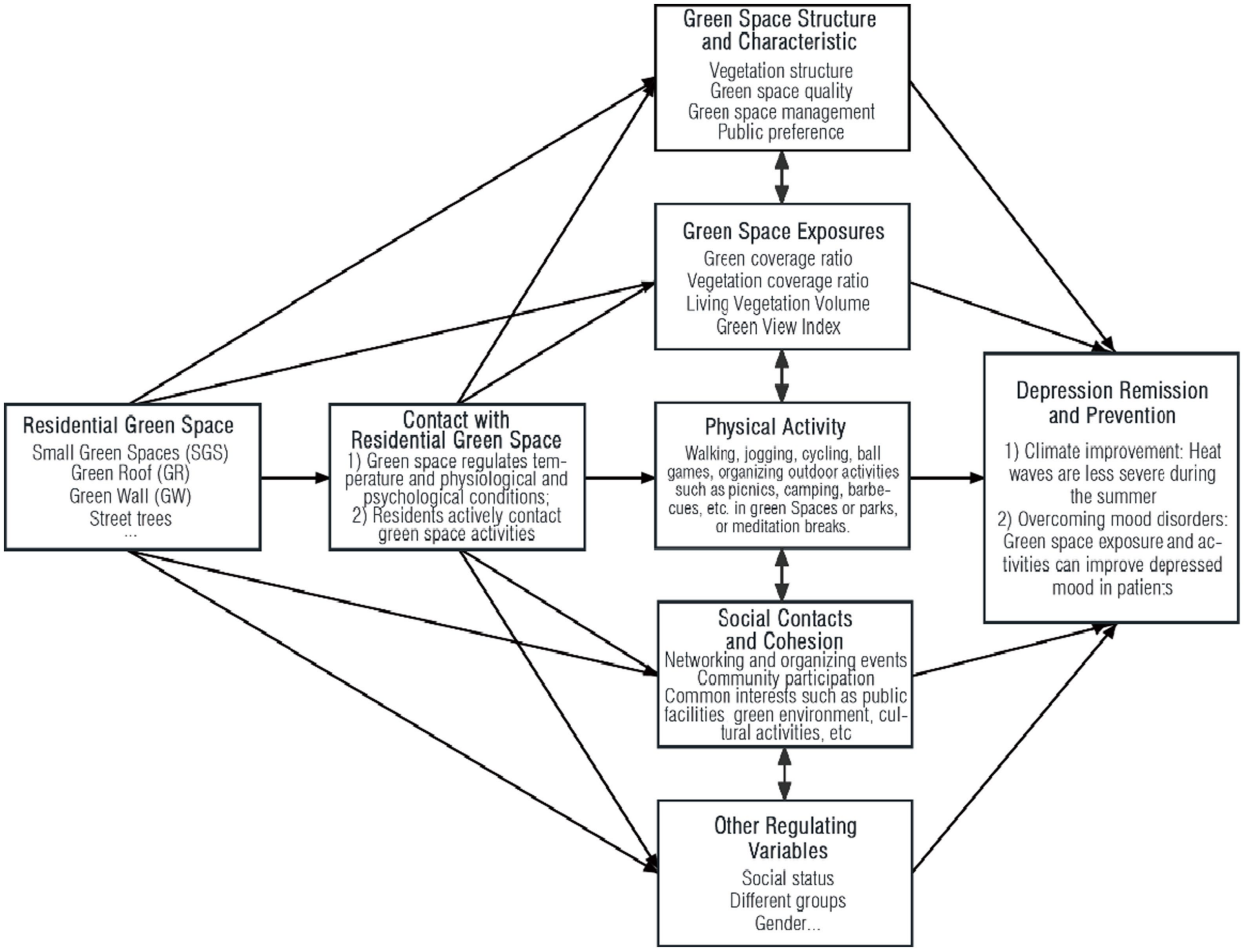
Conceptual framework of RGS cooling mechanisms and depression alleviation during extreme heat events’ impacting periods.

First, the specific framework (Figure 7) includes vegetation structure, green space quality, management, and public preference from the perspective of the composition and characteristics of the green space itself. These factors affect the mitigation impacts of green spaces on the heatwave environment and depression. These factors need to be evaluated and analyzed. The green index of RGS can be used to assess the cooling impact of green spaces on the heatwave environment and the alleviating effect of green space exposure on depression. In considering the composition and characteristics of green space itself, green space index, green space exposure, and other factors, the comprehensive impact of green space on heat wave environment and depression can be evaluated.

Second, the way of contact between residents and RGS and the social demand of residents for activity space in the residential community are also important factors affecting the alleviation of depressive symptoms by green space. This mainly represent residents’ social needs for the residential community’s activity space. It refers to their daily need to use green space for sports activities, social connections, and cohesion. With physical activities such as walking, jogging, leisure, socializing, and gardening, the social connection and cohesion of residents refer to the interaction and communication between them and others in the community, as well as the degree of identification and support for the common interests of the neighborhood (102). These factors can alleviate depressive symptoms by strengthening the individual’s social support network, promoting emotional communication and mutual help, and improving the sense of belonging and responsibility. Numerous studies have shown that social isolation and lack of social support are important factors leading to depression. In contrast, good social connection and cohesion can reduce feelings of social isolation, improve individual happiness and quality of life (103), thus, positively alleviate depressive symptoms. For example, community connection and participation can be established and strengthened through social and organized community activities. Community participation refers to residents’ active participation in community management, events, activities, and other affairs. In addition, it also represents common interests such as public facilities, a green environment, cultural activities, etc. (104). The higher the level of participation, identification, and support of residents for the common good, the stronger the social connection and cohesion which can alleviate depressive symptoms.

Finally, residents’ factors (personal social status, different population groups, age, etc.) also significantly affect the depression induced by heat exposure. Since individual’s social status includes education, income, occupation, different groups’ risks of depression vary. Studies have shown that people of lower social status are more susceptible to heat exposure (105, 106) which increases the risk of depression. For example, specific populations, including the older adult, pregnant women, and children, are particularly susceptible to the risks associated with heat exposure (107). These groups are prone to physical discomfort and depression, thus, increasing the risk of depression. Age is also an important factor in heat exposure-induced depression. With the increase of age, the body’s physiological function and metabolic capacity gradually decline, and the ability to tolerate high-temperature weather also weakens increasing the risk of depression. Therefore, residents’ factors are also critical to affect the different degrees of depression caused by heat exposure. Based on individual differences, various coping strategies need to be adopted such as strengthening the attention and protection of people with low social status, strengthening the health management of people susceptible to heat exposure, such as the older adult, pregnant women, and children, and raising the public awareness of heat exposure to reduce the risk of depression caused by heat exposure.

In summary, the following factors are combined: (1) climate improvement: the characteristics of green space reduce temperature and pressure sources; (2) overcoming mood disorders: exposure to green spaces and increased social and physical activity can improve depression; and (3) personal factors to the recovery of depressive symptoms.

### 4.2 Proposed methodology for multidimensional assessment and testing roadmap of RGS to reduce depression under extreme heat events

This section presents a multi-stage approach to evaluate the potential of RGS to mitigate depression exacerbated by extreme heat events. We propose using subjective and objective measurement tools to conduct multidimensional assessments of RGS characteristics. A longitudinal cohort study will track residents over time under heatwave conditions to analyze relationships between RGS factors, heat exposure, and depression. Comparative community interventions will evaluate RGS enhancements in one area versus a control neighborhood. Qualitative techniques like interviews and focus groups with depressed and non-depressed individuals will provide insights into lived experiences. This phased methodology combining quantitative and qualitative techniques can comprehensively investigate pathways for optimizing RGS to build resilience against heat-related mood disorders.

Currently, subjective measurement tools of residential environment characteristics are widely used in research. Several studies have used the Perceived Residential Environment Quality Indicators (PREQIs) developed and the Neighborhood Environment Walkability Scale (NEWS) (108, 109). The former is a more comprehensive evaluation of the living environment tool including physical assessment and social evaluation. The latter focuses on the walkability of the physical environment of the residential area and aims to measure residents’ perception of the attributes of the local climate (110). Objective measurement tools include street view data, field research data, GPS, GIS, RS, and other tools to investigate green space characteristics, accessibility, vegetation characteristics, and different levels (Table 2). The level of resident depression was objectively measured by the Center for Epidemiological Studies Depression Scale-10 (CESD-10) and the Patient Health Questionnaire-9 (PHQ-9). Previous studies have used these scales in large-scale epidemiological investigations around the globe and achieved good validity and reliability (100). Subjective measurement tools include the Self-rating Depression Scale (SDS), Beck Depression Inventory-II (BDI-II) (Table 2), etc. SDS is mainly used for adults, while BDI-II has no absolute restrictions on the population and is widely used in research (111, 112).

**Table 2:** Measurement tools and methods used to study green spaces in residential areas and depression.

**Categories**		**Measuring tools and methods**	**Analytical aspects**
Subjective measurement tools	RGS features	Residential subjective environmental quality questionnaire (PREQI)	Neighborhood lifestyle, environmental health/pollution, upkeep/care.
		Neighborhood environment walkability scale (NEWS)	Measure residents’ perceptions of local environmental attributes.
	Depression rating scale	Self-rating depression scale (SDS)	It is mainly used in adults and is difficult to assess in severe cases.
		Beck depression inventory-II (BDI-II)	High universality and wide use.
Objective measurement tools	RGS features	Field research data Street view data	Record the location, area, type, etc., of green space, photos, and videos.
		Global positioning system (GPS), Geographic information system (GIS), Remote sensing (RS)	Investigate the GCR, estimate the LVV, and obtain the green space structure and distribution.
	Depression rating scale	Center for epidemiological studies Depression scale-10 (CESD-10)	Generally suitable for outpatient visits by professionals such as psychiatrists or psychologists in medical institutions.
		Patient health questionnaire-9 (PHQ-9)	

**Figure 8:**
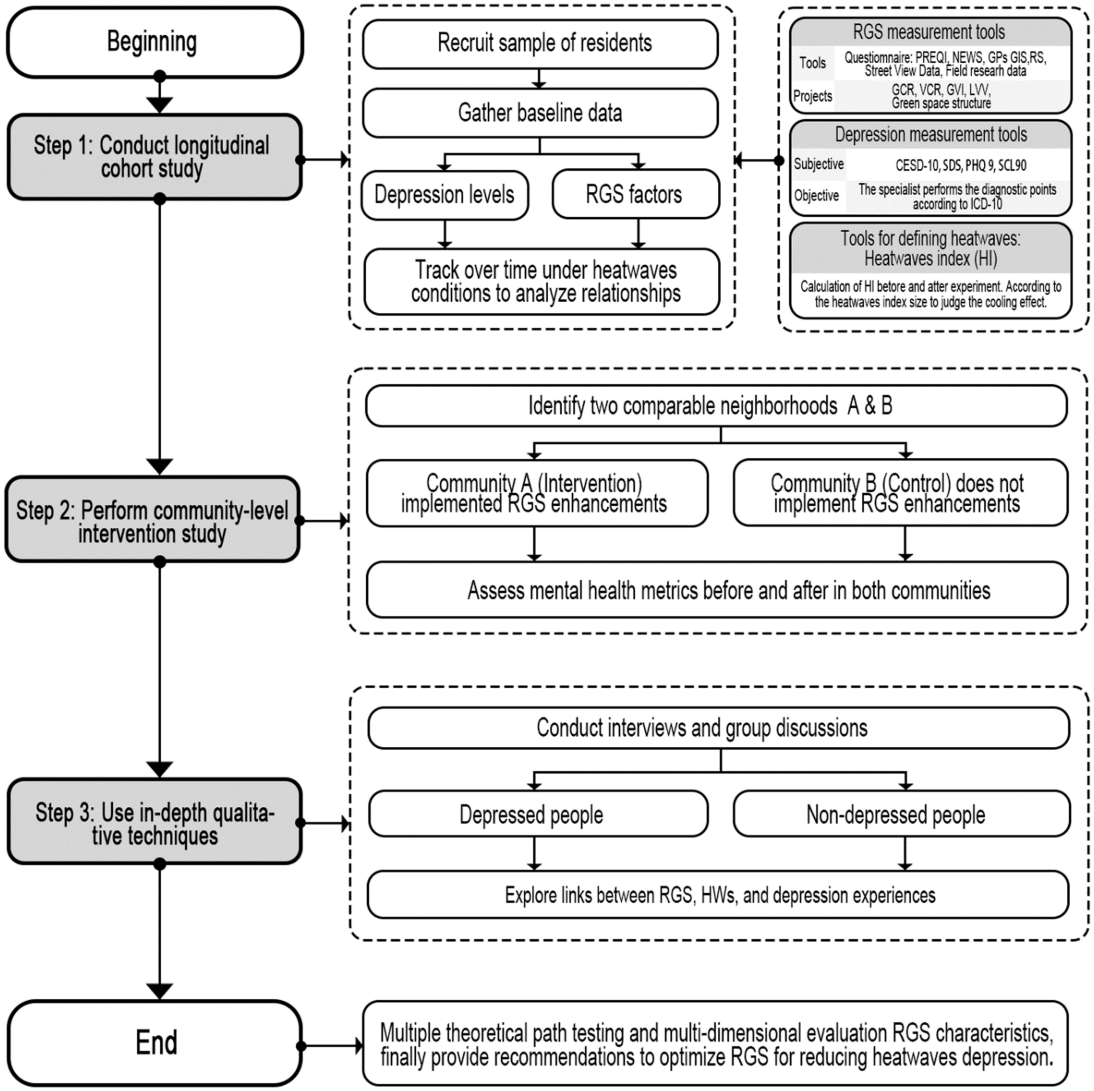
Hypothesis-testing roadmap of RGS for releasing depression under extreme heat events.

During the periods of extreme heat events, people experience heat stress, leading to various physical and psychological problems, which can be mediated by RGS. Therefore, designing a multidimensional evaluation-based RGS can help to reduce depression stress during heat waves (Figure 8). Various subjective and objective measurement tools above (Table 2) will be utilized for multi-faceted data collection on RGS factors and depression levels. We propose to conduct a longitudinal cohort study (113) that follows residents over time during heatwaves conditions in order to analyze the relationship between RGS indicators, heat exposure, and depression outcomes. Comparative community interventions will be implemented to evaluate RGS enhancements in one neighborhood area versus a control area without upgrades. This mixed methods research design integrating quantitative epidemiological and comparative studies with qualitative investigations can comprehensively elucidate pathways for optimizing RGS to strengthen resilience against climate change-related mental health burdens.

In step 1, we selected a certain number of residents for a longitudinal study, followed them up for a length of time, and collected data on their depressive symptoms and their residential landscape environment. It should be noted that the screened participants were all in the same climate environment under different follow-up periods. The initial step in this study involved determining the sample size and representativeness of the participants. Subsequently, we used standardized questionnaires or interview methods to collect data on the depressive symptoms of participants, and applied field surveys and remote sensing technology to collect data on their residential landscape environment.

Step 2 of the study was conducted in residential areas providing one group of residents having measures to improve the landscape environment, which was compared with another group of residents who were not offered mitigating measures. In this study, we selected two groups of residents in a residential area, one group received an intervention to improve the landscape environment, and the other group acted as a control group without any intervention. Through a questionnaire survey and on-site observation of residents in the two groups before and after the study, we compared indicators such as depressive symptoms, mental health level, and quality of life in the two groups to assess the impact of improving the landscape environment on reducing depressive symptoms.

In step 3, patients with depression and non-depressed patients were selected to conduct case studies in order to deeply understand their illness and the impact of the residential landscape environment on them and to explore the effects of the residential landscape environment on depression. In this stage of the study, we recruited a certain number of depressed patients and non-depressed patients, conducted a detailed investigation and assessment of their residential landscape environment, and thoroughly tried to understand the relationship between their illness and residential landscape environment through different research techniques such as semi-structured interviews and focus group discussions.

In conclusion, this phased methodology will generate robust evidence regarding if and how RGS may mitigate heatwave-associated depression through ecological and social mechanisms. The findings will inform planning and interventions for designing mentally restorative green spaces and climate-resilient neighborhoods. It is important to note that this is only a preliminary design framework, and numerous factors, to include local climate conditions, building types, and the surrounding environment, influenced the specific implementation. Therefore, adequate local research and investigation were required before implementing a design. Further feasibility studies are needed to tailor the testing roadmap before implementation in specific local contexts.

## 5 Conclusion

In reviewing the research on extreme heat events and depression, the CiteSpace analysis mainly focuses on “RGS landscape evaluation from the perspective of public health” and “mental health mechanism of RGS.” Given the accelerating effects of extreme weather on mental health, there are still conflicting knowledge gaps in the study of the impact of green space on depression, so studies should integrate the relationship between RGS, extreme heat events, and depression. It was found that the specific factors that influence RGS on depression under heatwave conditions are poorly studied, highlighted by the severity of the global heatwaves problem which has been inadequately studied. We reviewed the relationship between extreme heat events and depression, the perspective of RGS ecosystem services, and the health perspective of RGS and depression and found that different RGS factors (green types, indicators, and structures) were negatively correlated with temperature and depression. Providing ecosystem services through RGS involves multiple pathways that are beneficial to mental health, including the active cooling effect of green space and the activation of patients’ self-activity in contact with green space, as well as the passive regulation of psychology. Overall, our findings suggest that RGS may have the potential to be an effective intervention for people with depression, and further research in this area is warranted.

On this basis, we propose a conceptual framework for using RGS to alleviate depression during extreme heat events. We also summarized a measurement tool for residential green space and a measurement tool for depressive symptoms, as well as a test roadmap for RGS to reduce extreme heat events and reduce blood pressure based on multidimensional assessment as a basis for further research to optimize and design the relationship between RGS and depression in the context of extreme heat events.

In the future, it is necessary to establish a more scientific RGS health evaluation system, explore the potential mechanism of the green space environment’s impact on health through quantitative analysis, and give full play to the biodiversity effect of green space in alleviating depression in residential areas. Ultimately, this study contributes to a more systematic and comprehensive understanding of the relationship between RGS and depression and supports evidence-based policies and interventions to improve the mental health in the context of extreme heat events.

## Author contributions

YY: Conceptualization, Data curation, Formal analysis, Methodology, Writing - original draft, Writing - review & editing. YZ: Conceptualization, Funding acquisition, Methodology, Supervision, Validation, Writing - original draft, Writing - review & editing. SS: Supervision, Writing – review & editing.
